# Living Beyond 80: A Longitudinal Study of Cognitive and IADL Disablement Among the Oldest Mexican Americans

**DOI:** 10.1093/geroni/igab046.1846

**Published:** 2021-12-17

**Authors:** Jiwon Kim, Jacqueline Angel, Sunshine Rote

**Affiliations:** 1 UT Austin, Austin, Texas, United States; 2 UT Austin, UT Austin, Texas, United States; 3 University of Louisville, Louisville, Kentucky, United States

## Abstract

Mexican Americans live longer on average than other ethnic groups, but often with protracted cognitive and physical disability. Little is known, however, about the role of cognitive decline for transitions in IADL disability and tertiary outcomes of the IADL disablement for the oldest old. We employ the Hispanic Established Populations for the Epidemiologic Study of the Elderly (2010-2011, 2012-2013, 2016, N=1,078) to investigate the longitudinal patterns of IADL decline using Latent Transition Analysis. Three IADL groups were identified: independent (developing mobility limitations), emerging dependence (limited mobility and community activities), and dependent (limited mobility and household and community activities). Declines in cognitive function were a consistent predictor of greater IADL disablement, and loneliness was a particularly salient distal outcome for emerging dependence. These results highlight the social consequences of cognitive decline and dependency as well as underscore important areas of intervention at each stage of the disablement process.

